# Development of Groundwater Levels Dataset for Chile since 1970

**DOI:** 10.1038/s41597-023-02895-5

**Published:** 2024-02-05

**Authors:** Héctor Leopoldo Venegas-Quiñones, Rodrigo Valdés-Pineda, Pablo García-Chevesich, Juan B. Valdés, Hoshin V. Gupta, Martha P. L. Whitaker, Ty P. A. Ferré

**Affiliations:** 1 University of Arizona, Hydrology and Atmospheric Sciences, 1133 E James E. Rogers Way, Tucson, AZ 85719 USA; 2 Piteau Associates - Tetra Tech, Water Management Group, 2500 North Tucson Boulevard, Tucson, AZ 85716 USA; 3 WH2O. Association of Hydrologists and Hydrogeologists, Santiago, Chile; 4 Colorado School of Mines. Department of Civil and Environmental Engineering. 1500 Illinois St, Golden, CO 80401 USA; 5 Intergovernmental Hydrological Programme. United Nations Educational, Scientific, and Cultural Organization. Av. Julio Maria Sosa 300, Montevideo, Uruguay

**Keywords:** Hydrology, Hydrology

## Abstract

Access to accurate spatio-temporal groundwater level data is crucial for sustainable water management in Chile. Despite this importance, a lack of unified, quality-controlled datasets have hindered large-scale groundwater studies. Our objective was to establish a comprehensive, reliable nationwide groundwater dataset. We curated over 120,000 records from 640 wells, spanning 1970-2021, provided by the General Water Resources Directorate. One notable enhancement to our dataset is the incorporation of elevation data. This addition allows for a more comprehensive estimation of groundwater elevation. Rigorous data quality analysis was executed through a classification scheme applied to raw groundwater level records. This resource is invaluable for researchers, decision-makers, and stakeholders, offering insights into groundwater trends to support informed, sustainable water management. Our study bridges a crucial gap by providing a dependable dataset for expansive studies, aiding water management strategies in Chile.

## Background & Summary

Groundwater is a vital resource in Chile^1^ where its management faces multifaceted challenges. These encompass increasing flood risks, inefficient water use, leakages, unauthorized consumption, conflicts, and the constrained availability of data^2,3^. Moreover, over the past few decades, groundwater usage has surged, accompanied by diminishing storage capacity. This situation has led to over 20% of aquifers experiencing overexploitation due to the absence of sustainable management practices^4,5^.

Assessing and managing groundwater resources in Chile poses substantial hurdles, largely attributed to the scarcity of in-situ observations. This scarcity is especially pronounced in arid and semi-arid regions, where monitoring infrastructure is lacking. The resulting dearth of well measurements obstructs precise groundwater modeling and management^6,7^. Sufficient temporal and spatial data are imperative for accurate groundwater models to inform decision-making effectively^5,8^.

The General Directorate of Water Resources of Chile (DGA - dga.mop.gob.cl) is responsible of overseeing water rights while prioritizing environmental preservation. To facilitate this, the concept of Common Use Hydrogeological Sectors (SHACs *in Spanish*) has been adopted. SHACs delineate distinct aquifer portions with unique hydrological characteristics (Decree No. 203 of 2013^9^), enabling informed decision-making and sustainable groundwater exploitation assessment. Currently, Chile comprises 715 SHACs, with 109 designated as prohibited zones for new groundwater extraction.

Numerous studies in Chile have sought to evaluate different regions, but the challenge of obtaining comprehensive, reliable groundwater data remains consistent. Despite localized studies focusing on managing groundwater, acquiring precise data remains complex. In their study, Oyarzún *et al*. investigated the hydro chemical and isotopic traits within the Limarí River basin, situated in Chile’s arid North-Central region. Despite the existence of a relatively extensive monitoring network supervised by the DGA for assessing surface water quality, the authors underscore the limited coverage of groundwater data in both spatial and temporal dimensions, highlighting associated challenges^10^. In contrast, Chilean water users operate within a framework marked by inadequate monitoring of groundwater availability, deficient oversight, and insufficient data collection regarding groundwater levels^11^. This framework is compounded by a significant lack of knowledge concerning Chilean aquifers^12^. Consequently, evaluating any aquifer mandates the establishment of monitoring mechanisms and comprehensive data collection. These components serve as essential prerequisites for an effective assessment of aquifer conditions and characteristics.

The main objectives of this study are:

1. ***Inform decision-making***: The comprehensive groundwater dataset is aimed at offering policymakers, water managers, and researchers, reliable and current information for informed decision making^13,14^. The analysis of groundwater data is aimed to identify trends, enabling effective policies for sustainable water use and conservation^15,16^.
2. ***Enhance understanding and modeling***: The integration of varied datasets offers a holistic understanding of groundwater systems, incorporating geological, hydrological, climatic, and socio-economic aspects. The study also provides opportunities for the development of advanced models for groundwater availability predictions, identifying vulnerability, and assessing climate change and human impact^15,17,18^.
3. ***Foster stakeholder engagement*:** A centralized, accessible groundwater dataset is aimed to reinforce transparency, accountability, and collaboration among agencies, researchers, and communities^19,20^.
4. ***Facilitate long-term monitoring and assessment*:** The comprehensive dataset is aimed to establish a robust monitoring framework, identifying trends and changes in groundwater levels, water quality, and other factors, guiding proactive management and early warning^21,22^.

This dataset not only assists in the sustainable management of aquifers but also provides valuable information for large-scale studies and research endeavors^14,23,24^. The potential uses of the groundwater dataset are wide-ranging. It serves as a valuable resource for researchers studying groundwater management, climate change, and sustainable water resource planning^25,26^. Policymakers can derive evidence-based policies, and water managers can optimize allocation and conservation^27^. The dataset also supports local community education and awareness^28^. Thus, the development of this dataset addresses Chile’s groundwater management challenges, fostering informed decision-making, modeling, engagement, and long-term assessment. By leveraging this resource, Chile can achieve sustainable groundwater use, ensuring water security in the face of challenges. Moreover, by incorporating elevation data for each well station, the study aims to estimate water elevation—a critical value for assessing hydraulic head. This additional parameter enhances the dataset’s utility in understanding groundwater dynamics and contributes to a more comprehensive evaluation of aquifer conditions.

Currently, a centralized platform for the groundwater dataset is lacking, hindering automated access. Manual well-by-well retrieval is time-consuming and challenging for multiple well data extraction. This limitation highlights the need for user-friendly platforms that can provide streamlined access to the comprehensive groundwater dataset in Chile.

## Data and Methods

### Data sources

The groundwater level data for this study, encompassing depth to water (DTW) and water levels above sea level (GWL), were sourced from the DGA. A comprehensive request was made to acquire data from all available monitoring wells across the nation, spanning 1970 to present, covering both public and private wells. Each monitoring well’s dataset comprises vital parameters as: name, BNA code (including basin, sub-basin, and area), elevation above sea level, longitude, latitude, and instantaneous DTW values in meters. Additionally, the dataset includes a record status flag, reflecting the condition of each well during measurements. Six status categories are assigned: “Static,” “Dynamic,” “No Access,” “Dry,” “Embedded,” and “Surging”. This classification offers insights into well dynamics, contributing to a comprehensive national groundwater system understanding. Notably, the classification scheme preserves raw record originality and integrity, ensuring no modifications or omissions of the original dataset. This approach safeguards the purity of the dataset, upholding its value for end-users. Finally, all the data has been uploaded to the OSF (Open Science Framework) for accessibility and transparency^29^.

### Data processing and methods

The raw records sourced from the DGA were organized into two distinct formats, corresponding to different timeframes. Specifically, the dataset spanning 1970 to 2018 follows a specific format denoted as “***Pozos1970-2018.rar***” and it is accessible at: https://osf.io/pguw7. Conversely, data spanning 2019 to 2021 is structured differently and labeled as “***Pozos2019-2021.rar***” accessible at https://osf.io/qnzgu.

To address this divergence, we developed two Python algorithms (one for each format) to systematically manage and extract relevant information. This meticulous approach ensured precise and efficient data processing across several temporal periods. By accommodating these disparate formats, we seamlessly integrated historical and recent groundwater data into a unified dataset. This integration, facilitated by the file “***concatenated_files.zip***” available at https://osf.io/m5k72, provides a holistic perspective of the evolution of groundwater systems over time.

**Fig. 1:**
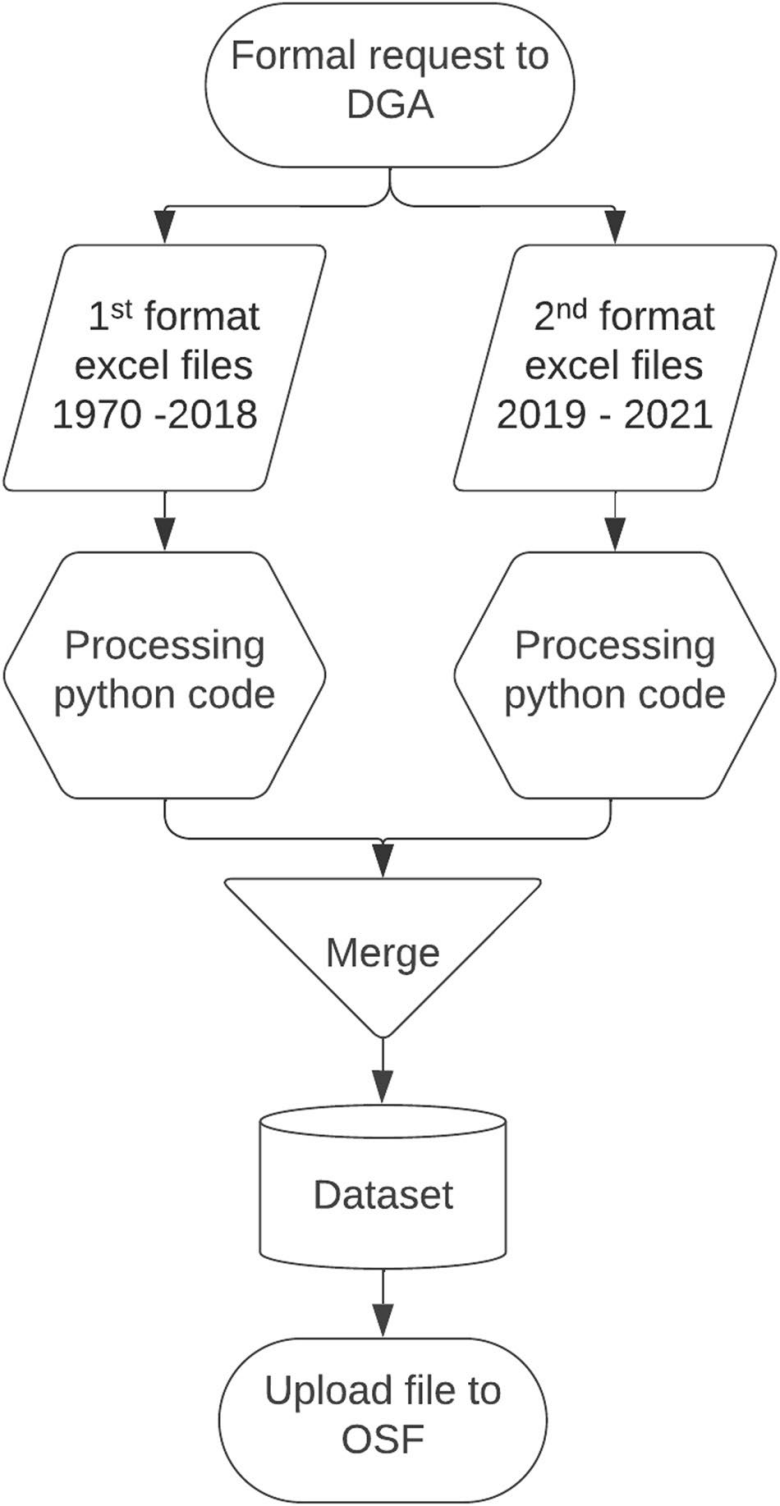
Establishing the national groundwater levels dataset for Chile: A flowchart detailing the processing steps, initiated with raw records from the DGA. It encompasses two formats: the first, “Pozos1970-2018.rar,” contains historical data (1970-2018), and the second, “Pozos2019-2021.rar,” has a different structure for the years 2019-2021.

In alignment with principles of transparency and openness, we have ensured that the python code used in the data processing is openly accessible. This code is available within the dataset under the filename “***processing.ipynb***” and can be accessed at https://osf.io/swdg9. By making this code publicly available, an invitation is extended to other parties to utilize it for their research, analysis, and decision-making efforts. This approach promotes inclusivity, collaborative endeavors, and the sharing of knowledge. A schematic flowchart shows the sequence of steps involved and the processing workflow utilized to create the groundwater dataset (Fig. 1).

The meticulous approach employed in developing these algorithms facilitates precise data processing across different temporal periods. Following the processing of both formats, the datasets are seamlessly integrated into a unified dataset using the file “concatenated_files.zip” available at https://osf.io/m5k72. This integration provides a holistic perspective of the evolution of groundwater systems in Chile over time.

In alignment with principles of transparency and openness, the python code used in the data processing is made publicly available to invite other parties to utilize it for their research, analysis, and decision-making efforts. This collaborative and inclusive approach aims to promote the sharing of knowledge and collaborative endeavors in groundwater research.

## Data Records

**Fig. 2:**
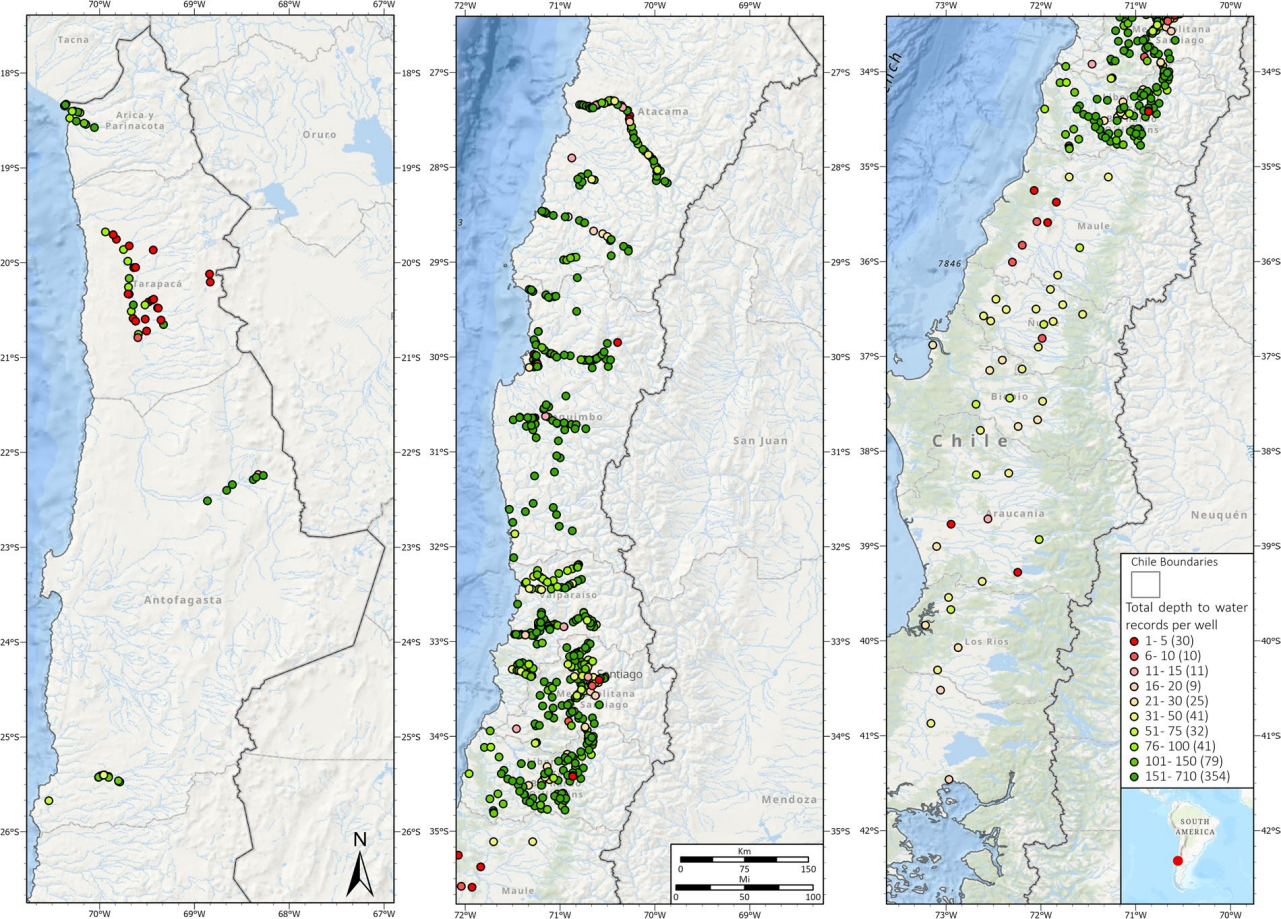
Geographical distribution of DTW records available for Chile, spanning from 1970 to 2021.

The dataset comprises a total of 1,356 Excel files. Among these, 854 adhere to the first format, while 502 align with the second format. Within these files, a substantial array of information is contained, encompassing a grand total of 122,720 DTW records, measured in meters. This extensive dataset contains readings from 635 wells distributed along Chile (see Fig. 2 for details). Notably, within this dataset, 319 wells exhibit an elevation reading of zero, while 316 wells display non-zero elevation values. The instances where the elevation is zero can be attributed to the absence of official data corresponding to the respective station. Additionally, it is important to mention that the raw data from the DGA includes negative values. While we believe these may be errors, we have included the entire dataset in its entirety for potential authors. It’s crucial to note that the negative values are not a result of the Python code but rather an attribute of the data provided by the DGA. The groundwater dataset for Chile comprises two main datasets, each with a specific file format:

1. Pozos1970-2018 dataset

- File type: Compressed RAR Archive
- File name convention: Pozos1970-2018.rar
- Description: This dataset contains groundwater level data spanning the period from 1970 to 2018. It includes records from monitoring wells located throughout Chile. These records provide essential information such as well name, BNA code (basin, sub-basin, and area), elevation above sea level, longitude, latitude, and instantaneous depth to water (DTW) values in meters. Each record also includes a status flag indicating the condition of the well during measurements, with categories like “Static,” “Dynamic,” “No Access,” “Dry,” “Embedded,” and “Surging.”
- Access link: Pozos1970-2018 dataset (https://osf.io/pguw7)

2. Pozos2019-2021 dataset

- File type: Compressed RAR archive
- File name convention: Pozos2019-2021.rar
- Description: This dataset contains groundwater level data for the years 2019 to 2021. It follows a different format compared to the Pozos1970-2018 dataset and includes records from various monitoring wells across Chile. The data includes well names, BNA codes, elevations above sea level, longitudes, latitudes, and instantaneous DTW values in meters. Like the previous dataset, it also includes a status flag indicating well condition during measurements.
- Access link: Pozos2019-2021 dataset (https://osf.io/qnzgu)

3. Concatenated files

- File type: Compressed ZIP archive
- File name convention: concatenated_files.zip
- Description: This ZIP file contains processed data resulting from the integration of the two datasets mentioned above. It ensures that historical and recent groundwater data are combined into a unified dataset for comprehensive analysis. The integrated data offer insights into the evolution of groundwater systems over time.
- Access link: Concatenated files (https://osf.io/jhvqd)

4. Python code for data processing

- File type: Jupyter Notebook (IPython)
- File name convention: processing.ipynb
- Description: This Jupyter Notebook contains the python code used for processing the groundwater level data from the two datasets. The code is openly accessible, encouraging other researchers and analysts to utilize it for their research, analysis, and decision-making efforts. It promotes inclusivity, collaboration, and knowledge sharing in groundwater research.
- Access link: Processing code (https://osf.io/swdg9)

5. Elevation datasets

- File type: External datasets
- Description: Two elevation datasets were incorporated into the groundwater dataset to enhance the quality and analytical capabilities of the data. These elevation datasets, namely NASADEM (Shuttle Radar Topography Mission data - a Digital Elevation Model, DEM)^30^ and ALOS PALSAR elevation data^31^ (a Digital Terrain Model, DTM), offer elevation values at 30 meters resolutions. They enable precise comparisons between groundwater levels and terrain, providing valuable insights into the spatial relationships between groundwater and the landscape.

## Technical Validation

To ensure the validity and quality of the groundwater level data in the dataset, standard statistical methods were employed for basic data quality assessment. The primary focus of this validation was to detect any anomalies or potential errors in the dataset. Key statistical metrics, such as the minimum, average, maximum, and standard deviation of the DTW records, were calculated and analyzed for each region within Chile.

**Fig. 3:**
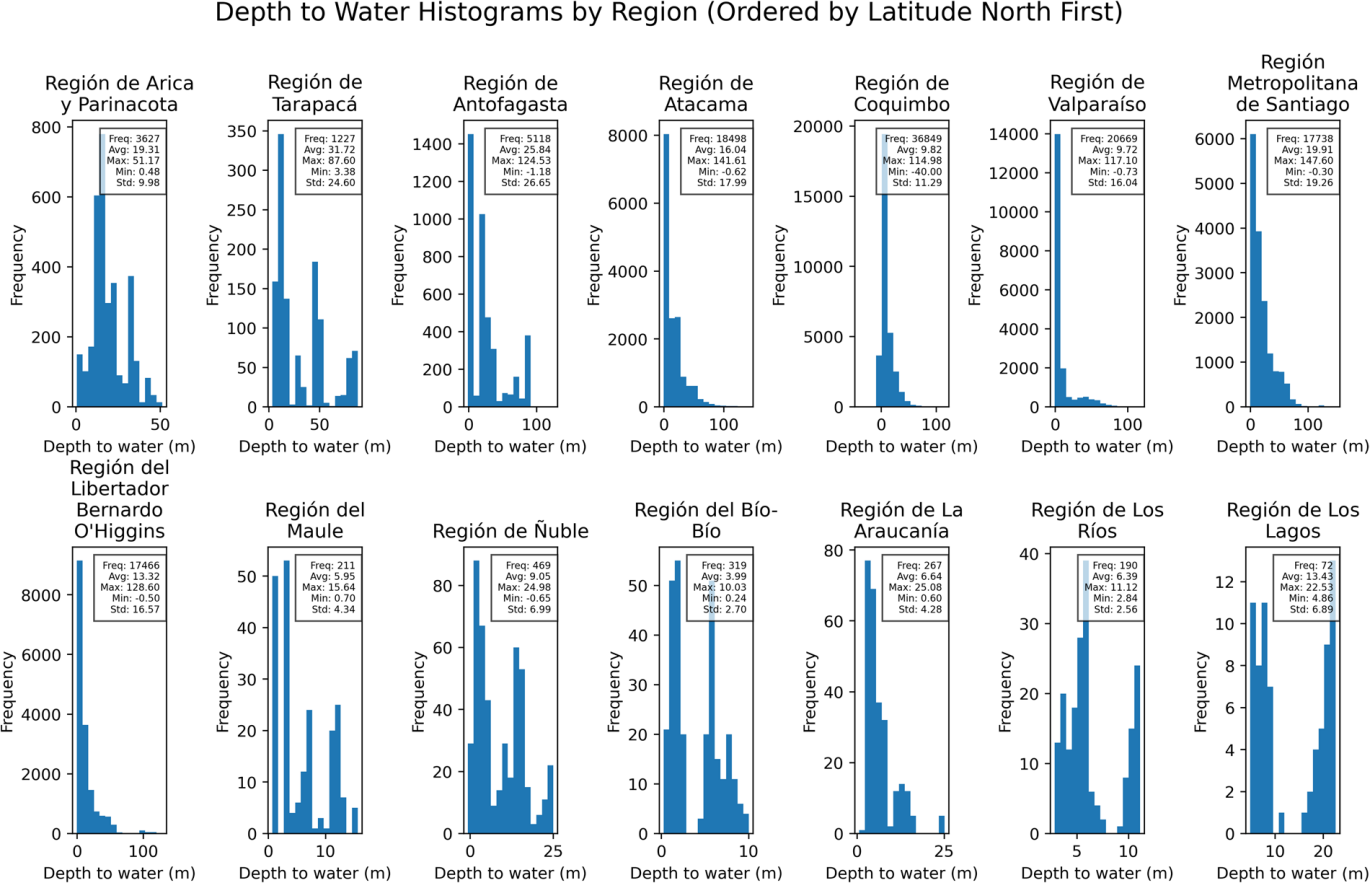
Frequency distribution of DTW measurements by region. The regions are organized by latitude.

The statistical analyses revealed valuable insights into the characteristics of the groundwater data while maintaining the integrity of the original dataset. The minimum and maximum DTW values provided a range of observed groundwater levels within each region. The average DTW values offered an understanding of the central tendency of groundwater levels, and the standard deviation quantified the variability or dispersion of the data. In the same context, Fig. 3 provides a visual representation of the frequency distribution of DTW measurements by region. The regions in Chile are organized by latitude, allowing for a clear geographical perspective of the groundwater data distribution. This frequency histogram divides the DTW dataset into 30 bins, each representing a range of DTW values.

By employing these standard statistical measures, the dataset was thoroughly validated without introducing any arbitrary modifications to the original groundwater records. This approach ensures that researchers and users can access the dataset with confidence, knowing that it has undergone a rigorous validation process. It also allows for unbiased and robust analyses by researchers who access the dataset for their specific studies, as no inadvertent biases or errors were introduced through arbitrary alterations of the groundwater records.

Additionally, a crucial stage involves the identification of outliers using z-scores, followed by the incorporation of this information into the dataset. Outliers, characterized by data points significantly deviating from the mean, often signify noteworthy irregularities or potential errors in the dataset. The z-score, a statistical metric, quantifies the extent to which a data point deviates from the mean in terms of standard deviations. The implemented function calculates z-scores for each data point in a given series by normalizing the deviation from the mean using the standard deviation. Subsequently, these z-scores are compared against a predefined threshold, conventionally set at 3 standard deviations. Notably, a new column is created in the dataset, marked with Boolean values (True or False), designating whether each data point is an outlier according to the established threshold. This column serves as a practical indicator, facilitating the automated recognition of extreme values within the dataset. The adaptability to adjust the threshold adds a layer of customization, enabling the fine-tuning of the outlier detection process to accommodate the specific characteristics and demands of the dataset.

**Fig. 4:**
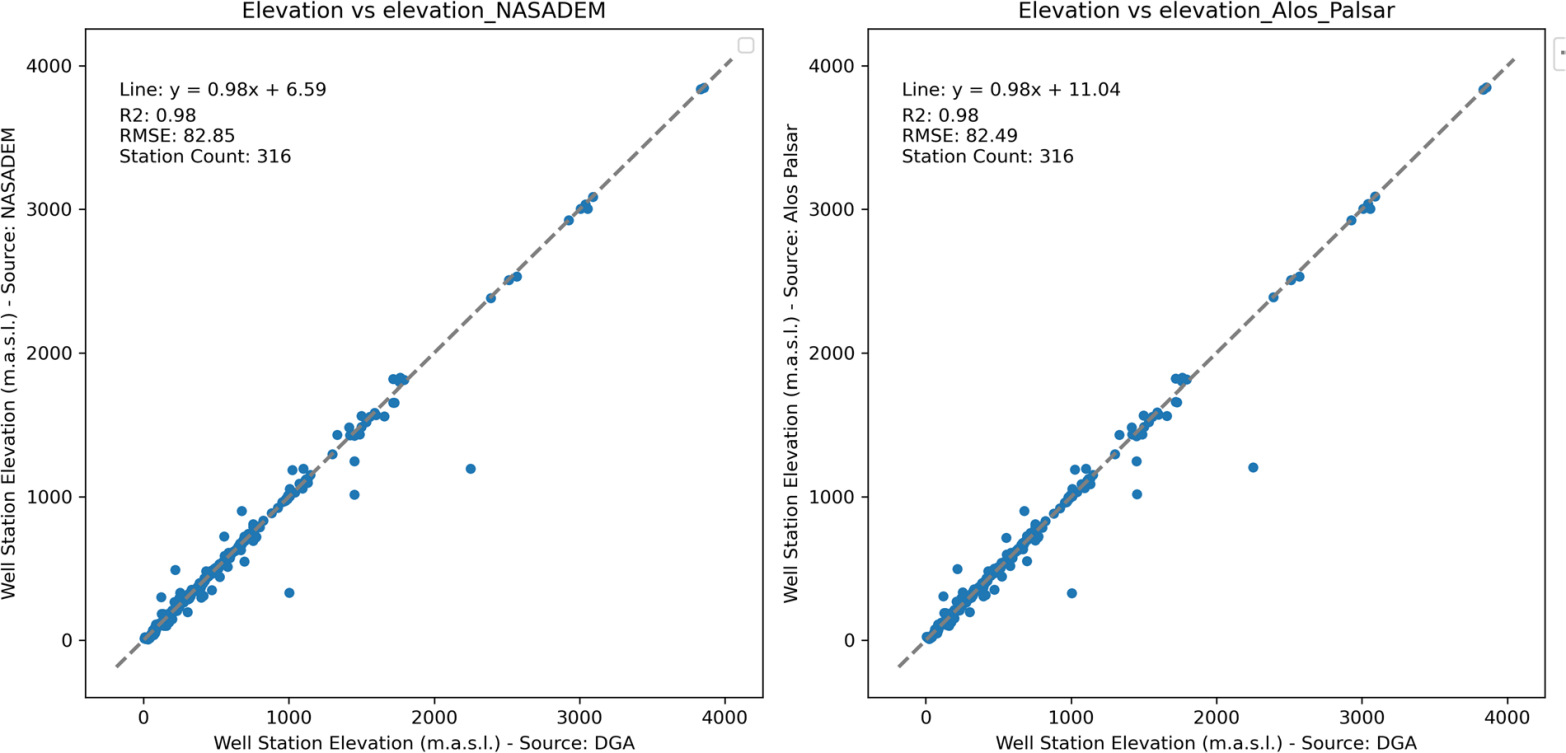
One-to-one scatter plot: Comparison of elevation data between Dirección General de Aguas (DGA) and remote sensing at well stations with elevations above 0 (316 out of 640).

In bolstering the robustness of our dataset, a secondary validation step involves a rigorous comparison of elevation data sourced from the DGA with datasets obtained from NASADEM and Alos Palsar. This dual validation strategy aims to enrich the dataset by integrating elevation information from diverse sources at well stations, fostering a more nuanced understanding of terrain and elevating the accuracy of groundwater elevation estimates. The comparative analysis serves as a crucial cross-validation mechanism, enabling the identification of any anomalies or discrepancies within the elevation dataset. The incorporation of NASADEM and Alos Palsar elevation data acts as an additional layer of assurance, ensuring the reliability of the groundwater level dataset. The harmonization observed among these disparate sources not only fortifies the overall quality of the dataset but also significantly enhances the precision of groundwater elevation estimations. This comprehensive validation approach adheres to scientific rigor, providing researchers with a dependable dataset for substantive investigations and analyses. Figure 4 presents a pivotal visualization in the form of a one-to-one scatter plot, depicting the elevation data from the DGA against remote sensing elevation at well stations where recorded elevations are higher than 0. Given the uncertainty in the elevation for the DGA when it is 0, we have plotted only the stations with an elevation higher than 0. This is intended to provide future authors with elevation values for the remaining stations. As observed, the elevation values from remote sensing data align well with locations having an elevation higher than 0.

The technical validation process focused solely on confirming the accuracy and reliability of the dataset, adhering to the principles of transparency and data integrity. Users are encouraged to utilize the dataset for their research, analysis, and decision-making efforts with the assurance of its quality and reliability. Further analyses and interpretations of the data are left to the discretion of users and researchers.

### Uncertainty and future needs

The potential uncertainties associated to the groundwater levels dataset of Chile can arise from different sources, such as the influence of nearby pumping wells whose impact on groundwater levels might not be fully understood in this study. Furthermore, uncertainties can also emerge from the accuracy and comprehensiveness of the national sampling program used to estimate groundwater levels.

## Data Availability

The code to process the DTW records is archived at the OSF repository: https://osf.io/swdg9

## References

[1] Vargas-Payera, S., Taucare, M., Pareja, C. & Vejar, J. Improving school children’s understanding of water scarcity with a co-produced book on groundwater in Central Chile. *Hydrogeology Journal*, 10.1007/s10040-023-02641-6 (2023). 10.1007/s10040-023-02641-6 PMC10240114 37361320

[2] Donoso, G., Lictevout, E. & Rinaudo, J.-D. in *Sustainable Groundwater Management: A Comparative Analysis of French and Australian Policies and Implications to Other Countries* (eds Jean-Daniel Rinaudo, Cameron Holley, Steve Barnett, & Marielle Montginoul) 481-509 (Springer International Publishing, 2020).

[3] Rinaudo J-D, Donoso G (2019). State, market or community failure? Untangling the determinants of groundwater depletion in Copiapó (Chile). International Journal of Water Resources Development.

[4] Duran-Llacer, I. *et al*. Lessons to Be Learned: Groundwater Depletion in Chile’s Ligua and Petorca Watersheds through an Interdisciplinary Approach. *Water* **12** (2020).

[5] Héctor LV-Q, Mark T, Pablo AG-C (2020). Water Scarcity Or Drought? The Cause And Solution For The Lack Of Water In Laguna De Aculeo. Water Conservation & Management (WCM).

[6] Montecino, H. C., Staub, G., Ferreira, V. G. & Parra, L. B. Monitoring Groundwater Storage in Northern Chile Based on Satellite Observations and Data Simulation. *Boletim de Ciências Geodésicas* **22** (2016).

[7] Donoso G (2021). Management of Water Resources in Agriculture in Chile and its Challenges. International Journal of Agriculture and Natural Resources.

[8] Venegas, Q. *et al*. Trend Analysis of Precipitation, Groundwater Level and Flow Rate Data by using Mann-Kendall and Sen’s Slope Estimator Statistical Tests in the Petorca Communer. *American Journal of Environmental Sciences* **15**, 10.3844/ajessp.2019.180.187 (2020)

[9] Públicas, M. d. O. (Biblioteca del Congreso Nacional de Chile, 2014).

[10] Oyarzún R (2015). A hydrogeochemistry and isotopic approach for the assessment of surface water–groundwater dynamics in an arid basin: the Limarí watershed, North-Central Chile. Environmental Earth Sciences.

[11] Nelson-Nuñez J, Walters JP, Charpentier D (2019). Exploring the challenges to sustainable rural drinking water services in Chile. Water Policy.

[12] Valdés-Pineda R (2014). Water governance in Chile: Availability, management and climate change. Journal of Hydrology.

[13] Kath J, Dyer FJ (2017). Why groundwater matters: an introduction for policy-makers and managers. Policy Studies.

[14] Condon LE (2021). Global Groundwater Modeling and Monitoring: Opportunities and Challenges. Water Resources Research.

[15] Cuthbert MO (2019). Global patterns and dynamics of climate–groundwater interactions. Nature Climate Change.

[16] Minea, I., Boicu, D., Amihăiesei, V. & Iosub, M. Identification of Seasonal and Annual Groundwater Level Trends in Temperate Climatic Conditions. *Frontiers in Environmental Science* **10** (2022).

[17] Swain S, Taloor AK, Dhal L, Sahoo S, Al-Ansari N (2022). Impact of climate change on groundwater hydrology: a comprehensive review and current status of the Indian hydrogeology. Applied Water Science.

[18] Döll P, Müller Schmied H, Schuh C, Portmann FT, Eicker A (2014). Global-scale assessment of groundwater depletion and related groundwater abstractions: Combining hydrological modeling with information from well observations and GRACE satellites. Water Resources Research.

[19] Pani A, Mishra P (2022). Policies and community participation for integrated natural resource management: a review of transdisciplinary perspective. Journal of Social and Economic Development.

[20] Lopez-Maldonado Y, Batllori-Sampedro E, Binder CR, Fath BD (2017). Local groundwater balance model: stakeholders’ efforts to address groundwater monitoring and literacy. Hydrological Sciences Journal.

[21] Ehsani, M. R. *et al*. 2019–2020 Australia Fire and Its Relationship to Hydroclimatological and Vegetation Variabilities. *Water* **12** (2020).

[22] Valdés-Pineda, R. *et al*. The Impact of a Lack of Government Strategies for Sustainable Water Management and Land Use Planning on the Hydrology of Water Bodies: Lessons Learned from the Disappearance of the Aculeo Lagoon in Central Chile. *Sustainability* **14** (2022).

[23] Zeng B, Zhang Z, Yang M (2018). Risk assessment of groundwater with multi-source pollution by a long-term monitoring programme for a large mining area. International Biodeterioration & Biodegradation.

[24] Roy SS, Rahman A, Ahmed S, Shahfahad, Ahmad IA (2020). Alarming groundwater depletion in the Delhi Metropolitan Region: a long-term assessment. Environmental Monitoring and Assessment.

[25] Scanlon BR (2023). Global water resources and the role of groundwater in a resilient water future. Nature Reviews Earth & Environment.

[26] Wu W-Y (2020). Divergent effects of climate change on future groundwater availability in key mid-latitude aquifers. Nature Communications.

[27] Bojórquez-Tapia LA, Cruz-Bello GM, Luna-González L, Juárez L, Ortiz-Pérez MA (2009). V-DRASTIC: Using visualization to engage policymakers in groundwater vulnerability assessment. Journal of Hydrology.

[28] Thornton T, Leahy J (2012). Trust in Citizen Science Research: A Case Study of the Groundwater Education Through Water Evaluation & Testing Program1. JAWRA Journal of the American Water Resources Association.

[29] Venegas-Quiñones HL (2022). Open Science Framework.

[30] Farr, T. G. *et al*. The shuttle radar topography mission. *Reviews of geophysics* **45** (2007).

[31] Shimada M, Ozawa T, Fukushima Y, Furuya M, Rosenqvist A (2008). Japanese L‐Band Radar Improves Surface Deformation Monitoring. Eos, Transactions American Geophysical Union.

